# Folding driven self-assembly of a stimuli-responsive peptide-hyaluronan hybrid hydrogel

**DOI:** 10.1038/s41598-017-06457-9

**Published:** 2017-08-01

**Authors:** Robert Selegård, Christopher Aronsson, Caroline Brommesson, Staffan Dånmark, Daniel Aili

**Affiliations:** 1 Division of Molecular Physics, Department of Physics, Chemistry and Biology, Linköping University, SE-583 36 Linköping, Sweden; 2 Division of Molecular Surface Physics and Nanoscience, Department of Physics, Chemistry and Biology, Linköping University, SE-583 36 Linköping, Sweden

## Abstract

Protein-metal ion interactions are ubiquitous in nature and can be utilized for controlling the self-assembly of complex supramolecular architectures and materials. Here, a tunable supramolecular hydrogel is described, obtained by self-assembly of a Zn^2+^-responsive peptide-hyaluronic acid hybrid synthesized using strain promoted click chemistry. Addition of Zn^2+^ triggers folding of the peptides into a helix-loop-helix motif and dimerization into four-helix bundles, resulting in hydrogelation. Removal of the Zn^2+^ by chelators results in rapid hydrogel disassembly. Degradation of the hydrogels can also be time-programed by encapsulation of a hydrolyzing enzyme within the gel, offering multiple possibilities for modulating materials properties and release of encapsulated species. The hydrogel further shows potential antioxidant properties when evaluated using an *in vitro* model for reactive oxygen species.

## Introduction

Smart soft materials have received a growing interest due to their ability to change properties in response to environmental stimuli^1–5^. Responsive hydrogels in particular are widely used in applications such as tissue engineering^6^, controlled release^7, 8^ cell therapy^9^, 3D cell culture^10^, and bioprinting^11^. Hydrogels that respond to pH^12^, temperature^13^, light^14, 15^, enzymatic interactions^16^, and metal ions^17^, have been reported. Protein-metal ion interactions are ubiquitous in nature and can induce conformational changes in proteins and trigger assembly of complex supramolecular architectures^18, 19^. Several peptides that fold as a result of metal ion coordination have been designed de novo, offering robust alternatives to proteins in soft materials^20–22^. Self-assembly of peptide-based hybrid hydrogels controlled by metal ion coordination is thus an interesting strategy that could facilitate development of responsive hydrogels with tunable properties. In addition, metal ions such as Zn^2+^ show antimicrobial properties^23^ and promotes wound healing^24^. Zn^2+^ loaded hydrogels are thus of large interest for a wide range of biomedical applications.

Hyaluronic acid (HA), or hyaluronan, is one of the main components in the extracellular matrix and regulates numerous cellular processes and due to its biocompatibility, biodegradability and good gel-forming properties, frequently used as a component in hydrogels for biomedical applications^25–27^. Degradation of HA by reactive oxygen species (ROS) has been linked to several diseases and conditions with an inflammatory component, including rheumatoid arthritis and chronic wounds^28^. Interestingly, low molecular weight HA has been reported to exert antioxidant activity, which is more pronounced upon coordination of metal ions, such as Zn^2+^ and Cu^2+^ ^29–31^.

Herein, we report on the development of a responsive physical hybrid hydrogel formed as a result of Zn^2+^-induced peptide folding and dimerization of peptides conjugated to low molecular weight HA. The peptide (JR2E) is designed to fold into a helix-loop-helix motif and homodimerize into four-helix bundles in the presence of Zn^2+^ ^32^. By conjugating the peptide to HA the self-assembly, disassembly and rheological properties of the hydrogels can be tuned by the concentration of Zn^2+^, metal ion chelators, and by encapsulation of hydrolytic enzymes in the hydrogels, offering a novel strategy for obtaining responsive HA-based physical hydrogels. Furthermore, an *in vitro* model with isolated neutrophil granulocytes was used to evaluate the effect of the hydrogel, including the role of Zn^2+^, on the production of ROS.

## Results and Discussion

**Figure 1:**
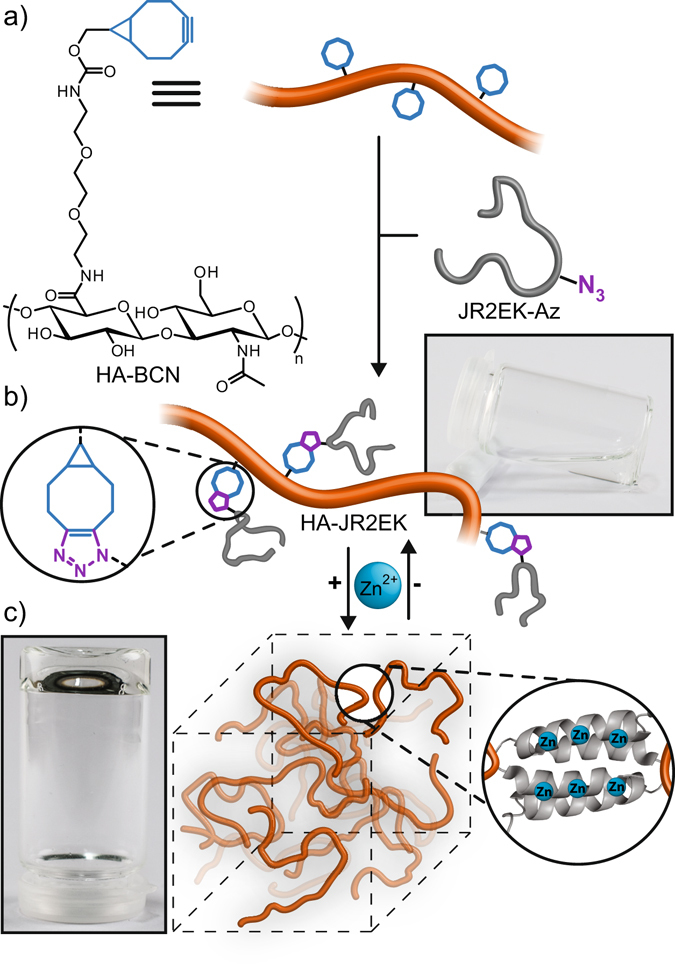
(**a**) Molecular structure and schematic illustration of HA-BCN that, when combined with the peptide JR2EK-Az, undergoes a SPACC reaction forming the responsive hybrid material HA-JR2EK. (**b**) The peptide component in HA-JR2EK exists as a random coil until addition of Zn^2+^ which causes it to fold and dimerize, resulting in a supramolecular cross-linking and self-assembly of a hydrogel (**c**). Removal of Zn^2+^ returns the peptides in HA-JR2EK to a random coil state and triggers disassembly of the hydrogel. Photographs of 2.5 wt % HA-JR2EK without (top) and with (bottom) 10 mM Zn^2+^.

To conjugate the peptides to the HA backbone, HA was first modified with bicyclo[6.1.0]nonyne (BCN) to support a strain promoted azide-alkyne cycloaddition (SPAAC) reaction^33^. The resulting HA-BCN had a degree of modification of ~7% based on ^1^H-NMR signals originating from the BCN group (Figure S1). The final peptide-polymer hybrid (HA-JR2EK) was obtained by conjugating the azide containing Zn^2+^-responsive peptide JR2EK-Az to HA-BCN via a SPAAC reaction (Fig. 1). The azide moiety in JR2EK-Az was included at position 22, located in the peptide loop region, in order to minimize the influence of conjugation on peptide dimerization and folding. ^1^H-NMR of HA-JR2EK confirmed the derivatization with a degree of modification of ~4% based on the ^1^H-NMR signals originating from histidine and phenylalanine residues in the peptide (Figure S1). ATR FT-IR further confirmed the derivatization by the evolution of strong amide I and II peaks (Figure S2). Consequently, ~10–15 JR2EK peptides were conjugated to each HA polymer resulting in a hybrid system with ~2:1 w/w ratio of HA:peptide.

**Figure 2:**
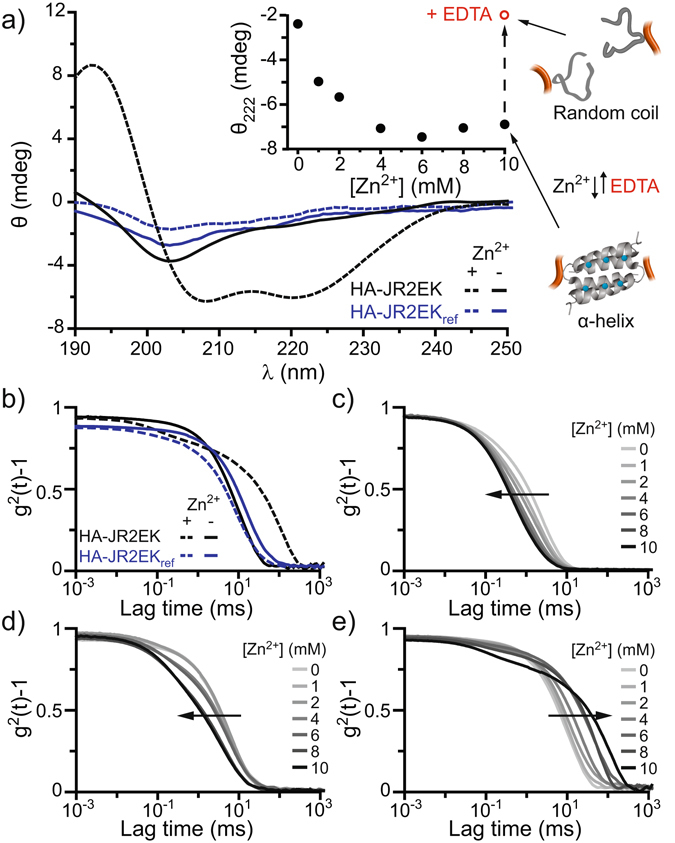
CD and DLS characterization of hydrogel formation induced by Zn^2+^. CD spectra of 0.1 wt % HA-JR2EK and HA-JR2EK_ref_ with and without 10 mM Zn^2+^. Inset: Increase in helicity of 0.1 wt % HA-JR2EK (at 222 nm) as a function of increasing [Zn^2+^]. The peptide unfolded upon addition of 10 mM EDTA. (**b**) Normalized autocorrelation functions for 1.0 wt % HA-JR2EK and HA-JR2EK_ref_ with and without 10 mM Zn^2+^. Normalized autocorrelation functions for 0.1 (**c**), 0.5 (**d**) and 1.0 (**e**) wt % HA-JR2EK with increasing [Zn^2+^].

Aqueous solutions (pH 7) of 2.5 wt % HA-JR2EK self-assembled immediately into hydrogels upon addition of Zn^2+^, as confirmed by tabletop rheology (Fig. 1 and Supplementary Movie 1). To investigate the role of peptide folding on hydrogelation, a second peptide-polymer conjugate was synthesized using a peptide with identical primary sequence as JR2EK-Az but having all *L*-alanine amino acid residues exchanged by *D*-alanine. This peptide, JR2EK_ref_, is thus unable to fold and dimerize^34^. The secondary structure of the HA conjugated peptides was characterized by circular dichroism (CD) spectroscopy. In the absence of Zn^2+^, the peptides in HA-JR2EK were random coils. Addition of Zn^2+^ resulted in CD spectra with characteristic minima at 208 and 222 nm, indicating that the peptides adopted an α-helical conformation (Fig. 2a). A gradual increase in helicity was seen when increasing the concentration of Zn^2+^, reaching saturation at 4 mM. Removal of the Zn^2+^ by the chelating agent EDTA lead to unfolding of the conjugated peptides (Fig. 2a inset) and disassembly of the hydrogels (Supplementary Movie 2). Because of the mix of *L*- and *D*-amino acid residues in JR2EK_ref_, HA-JR2EK_ref_ showed a weaker CD signal than HA-JR2EK even though both conjugates had the same degree of derivatization (Figure S1). HA-JR2EK_ref_ showed no ordered secondary structure in the absence nor in the presence of Zn^2+^. Neither HA nor HA-BCN responded to Zn^2+^ (Figure S3), confirming that only the conjugate with JR2EK undergoes a conformational change when exposed to Zn^2+^.

For the peptide-polymer conjugate to assemble into a hydrogel, the peptide must not only fold but also dimerize in order to link the individual macromolecules into a supramolecular network. The assembly of networks was investigated using dynamic light scattering (DLS) (Fig. 2b). Addition of Zn^2+^ to 1.0 wt % HA-JR2EK resulted in slower solution dynamics seen as an increase in lag times, strongly indicating assembly of larger networks. In contrast, addition of Zn^2+^ to HA and HA-JR2EK_ref_ resulted in shorter lag times, probably due to a certain degree of intramolecular coordination of Zn^2+^ via carboxylate groups in the HA backbone reducing the radius of gyration of the polymers (Figs 2b and S4). To further characterize the self-assembly process, samples with 0.1, 0.5 and 1.0 wt % HA-JR2EK were subjected to 0–10 mM Zn^2+^ (Fig. 2c–e). At 0.1 and 0.5 wt % the peptide-polymer conjugates contracted with increasing concentrations of Zn^2+^, most likely due to a certain amount of HA-Zn^2+^ interactions and intramolecular dimerization of the peptides, resulting in denser individual clusters of molecules. However, at 1.0 wt % the critical gelling concentration was reached and the probability of forming intermolecular networks predominated, resulting in a loosely associated hydrogel network.

**Figure 3:**
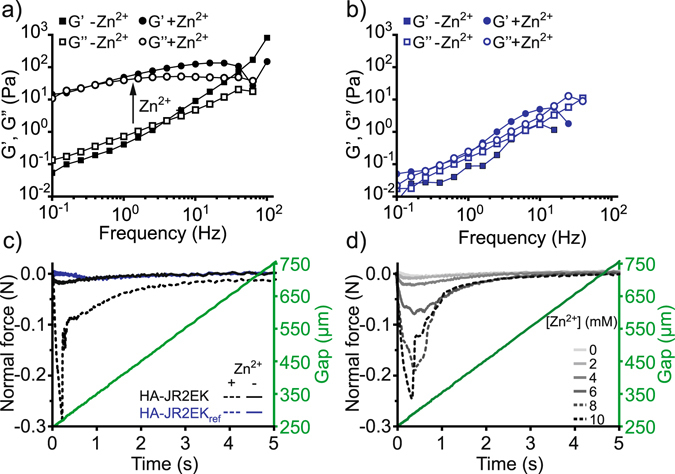
Rheological characterization of hydrogel formation induced by Zn^2+^. Rheological frequency sweeps of 2.5 wt % HA-JR2EK (**a**) and HA-JR2EK_ref_ (**b**) with and without 10 mM Zn^2+^. (**c**) Probe tack tests of 2.5 wt % HA-JR2EK and HA-JR2EK_ref_ with and without 10 mM Zn^2+^. (**d**) Probe tack test of 2.5 wt % HA-JR2EK with increasing [Zn^2+^].

In order to study the mechanical properties of the peptide-polymer conjugates and the hydrogels, we increased the concentration to 2.5 wt % and performed rheological frequency- and strain sweeps (Figs 3 and S5, respectively). At this concentration HA-JR2EK self-assembled into a soft viscoelastic hydrogel directly after addition of Zn^2+^ (Fig. 3a), whereas the apo-material behaved as a liquid. No significant differences could be detected in the rheological properties of HA-JR2EK_ref_ before and after addition of Zn^2+^ (Fig. 3b). It is thus clear that folding of the peptides is the main driving force for the assembly of the hydrogels. In addition, the self-assembled HA-JR2EK showed typical characteristics of supramolecular hydrogels, such as self-heling (Figure S6) and shear-thinning (Figure S7) properties.

Since the hydrogels were fairly sticky (Supplementary Movie 1), we examined the adhesive properties of HA-JR2EK and HA-JR2EK_ref_ when exposed to 10 mM Zn^2+^ using a probe tack test. Consistent with the hydrogel self-assembly process, only HA-JR2EK with Zn^2+^ showed any adhesive properties, seen as a significant negative normal force when retracting the probe from the sample (Fig. 3c,d). Furthermore, the adhesive bond could repeatedly be disrupted and reformed, once again showing the self-healing properties of the hydrogel (Figure S8). To investigate the nanoscale morphology of the hydrogels, HA-JR2EK was imaged using scanning electron microscopy (SEM) in the absence and presence of Zn^2+^. The hydrogels showed no fibrous structures indicating that the self-assembly occurs in a non-directional process resulting in an amorphous material (Figure S9). Since the samples had to be fixated using glutaraldehyde prior to imaging no significant differences could be seen between samples with and without 10 mM Zn^2+^.

**Figure 4:**
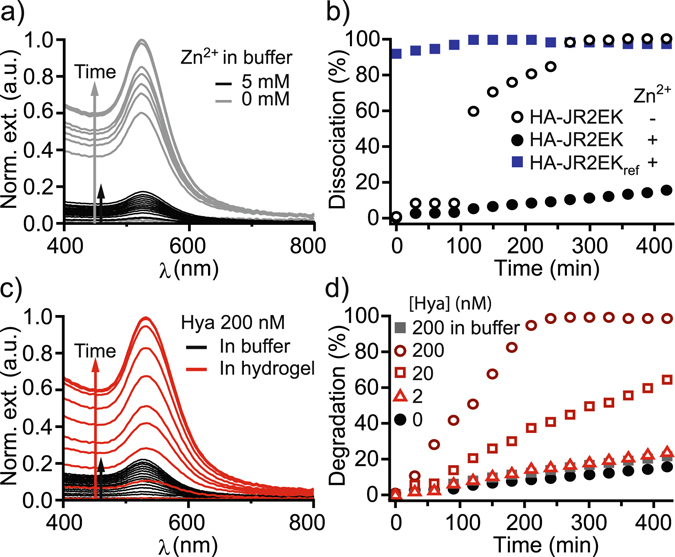
Dissociation and enzymatic degradation of hydrogels over time visualized by release of incorporated AuNPs. (**a**) UV-vis spectra of HA-JR2EK with JR2EC-AuNPs encapsulated within the hydrogel to visualize dissociation over time (measured every 30 min for 7 hours), in a buffer with and without 5 mM Zn^2+^. (**b**) Dissociation over time of hydrogels consisting of HA-JR2EK with and without 5 mM Zn^2+^ in the buffer and HA-JR2EK_ref_ with Zn^2+^ in the buffer. (**c**) UV-vis spectra of HA-JR2EK with 200 nM hyaluronidase (Hya) in the buffer or encapsulated within the hydrogel in a Zn^2+^ containing buffer. (**d**) Enzymatically catalyzed degradation over time of HA-JR2EK hydrogels with 0–200 nM hyaluronidase encapsulated within the hydrogel or 200 nM outside the hydrogel. HA-JR2EK and HA-JR2EK_ref_ concentrations were 2.5 wt % in all experiments and hydrogels were assembled using 10 mM Zn^2+^.

To aid in the visualization of the hydrogels as they are completely transparent, gold nanoparticles (AuNPs) decorated with the same Zn^2+^-responsive peptide were introduced into the hydrogels. Peptide immobilization on to the AuNPs was achieved by replacing the azide-modified lysine by a cysteine residue, to enable a thiol-Au coupling. The peptide-functionalized AuNPs (JR2EC-AuNPs) self-assemble reversibly upon addition of Zn^2+^ as a consequence of peptide folding and a dimerization-mediated bridging of the AuNPs^32^. Since the peptides immobilized on the AuNPs are available and can dimerize with peptides conjugated to HA, the JR2EC-AuNPs can be incorporated in the hydrogels during the hydrogel self-assembly process. The JR2EC-AuNPs were mixed with HA-JR2EK prior to addition of Zn^2+^. Addition of Zn^2+^ resulted in self-assembly of hydrogels containing homogeneously distributed AuNPs (Figure S10). Absence of nanoparticle aggregation, as indicated by the position of the localized surface plasmon resonance (LSPR) band (λ_max_ = 525 nm)^32^, strongly indicate that the immobilized JR2EC interact and dimerize with the HA conjugated JR2EK in the hydrogels. By monitoring the intensity of the AuNP LSPR peak in the solution above the hydrogels we could visualize hydrogel disassembly caused by Zn^2+^ depletion since the JR2EC-AuNPs were released (Fig. 4a, see also Figure S10). When submerged into a buffer without Zn^2+^, the hydrogel disassembled with a burst-like dissociation profile after about 2 hours incubation. However, if Zn^2+^ was present in the buffer the hydrogel remained stable with only minor nanoparticle release over an extended time period (Fig. 4a,b). HA-JR2EK_ref_ could not retain any JR2EC-AuNPs under any conditions.

Furthermore, we investigated if the degradation of the hydrogels could be controlled by subjecting them to hyaluronidase (Hya). Hya is an enzyme that hydrolyses the 1,4-linkages between *N*-acetyl-β-D-glucosamine and D-glucuronate in the HA backbone. Only minor degradation was seen when Hya (200 nM) was present in the buffer, indicating that diffusion of the enzymes into the hydrogel was very limited. On the other hand, when the same amount of Hya was encapsulated within the hydrogel a rapid degradation occurred (Fig. 4c). By varying the concentration of encapsulated Hya, the rate of hydrogel degradation could be tuned (Fig. 4d, see also Figure S11). The modest broadening and small red shift of the LSPR band indicate that residual HA-JR2EK was still attached to the released JR2EC-AuNPs, preventing extensive aggregation of the AuNPs, which would otherwise occur due to the Zn^2+^ content in the buffer (Fig. 4c)^32^.

**Figure 5:**
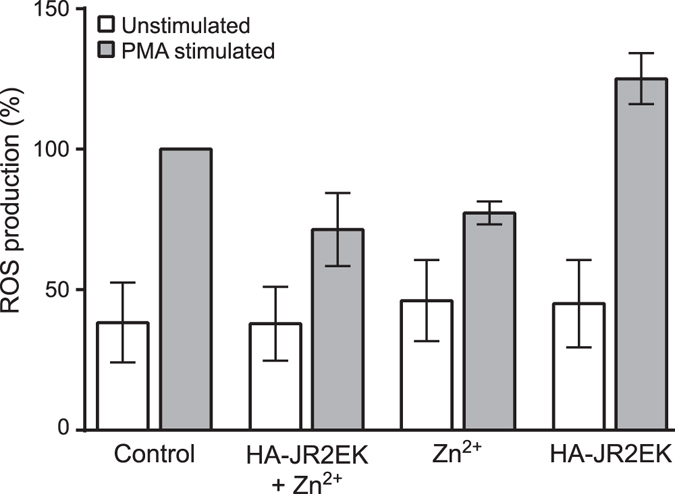
ROS modulatory effects of hydrogels. DCF-DA loaded isolated human neutrophil granulocytes were treated with ± HA-JR2EK (2.5 wt %) ± Zn^2+^ (10 mM) prior to PMA (1 µM) activation. Results are expressed as % ROS production with respect to PMA control ± S.E.M.

To evaluate the impact of the hydrogel system and its components on ROS production, an *in vitro* model with neutrophil granulocytes isolated from human whole blood was used. Neutrophil granulocytes are reactive immune cells that produce large amounts of ROS upon activation^35^. As a positive control and to induce ROS formation, the cells were stimulated with phorbol 12-myristate 13-acetate (PMA) (1 µM). The presence of Zn^2+^, alone or in combination with HA-JR2EK showed a moderate modulatory effect on the production of ROS as compared to the control, however, not statistically significant in the present setup (Fig. 5). Treatment with HA-JR2EK alone prior to PMA stimulation did not induce a decrease in ROS production, suggesting that the Zn^2+^ could play a role in the underlying mechanism although this requires further studies to be confirmed. Controls without addition of PMA confirmed that none of the components provoked a ROS response on their own as compared to resting control cells.

## Conclusions

In conclusion, we have developed a peptide-hyaluronan hybrid that self-assembles into soft and adhesive hydrogels as a consequence of Zn^2+^-induced folding and dimerization of the peptides. The mechanical properties, assembly and disassembly of the hydrogels could be tuned by the concentration of available Zn^2+^. We further demonstrate that the degradation of the hydrogels could be time-programed by encapsulating different amounts of hyaluronidase in the hydrogels. The Zn^2+^ containing hydrogel system also showed a potential ROS modulatory effect in line with previously published observations^30, 31^. Combined, this results in a very dynamic and bioinspired hydrogel system providing unique possibilities to modulate materials properties and control the release of encapsulated species.

## Experimental section

### General remark

All chemicals were purchased from Sigma Aldrich unless otherwise noted and used without further purification.

### Peptide synthesis

The peptides JR2EK (H2N-NAADLEKAIEALEKHLEAKGPKDAAQLEKQLEQAFEAFERAG-COOH), JR2EK_ref_ (L-Ala substitute with *D*-Ala) and JR2EC (Lys-22 exchanged for Cys) were synthesized on a Quartet automated peptide synthesizer (Protein Technologies, Inc.) using standard fluroenylmethoxycarbonyl (Fmoc) chemistry. Peptide synthesis was performed on a 0.1 mmol scale using Fmoc-Gly-Wang resin (Iris Biotech GmbH) as solid support and each amino acid (0.4 mmol, Iris Biotech GmbH) was coupled with *O*-(7-benzotriazole-1-yl)-1,1,3,3-tetra-methyluronium tetra-fluoroborate (TBTU) (0.4 mmol, Iris Biotech GmbH) as activator and diisopropylamine (DIPEA) (0.8 mmol) as base. Deprotection of Fmoc groups was accomplished by treatment with piperidine (20% in DMF, v/v, Applied Biosystems). To allow for a site specific incorporation of an azide moiety in the peptides loop regions of JR2EK and JR2EK_ref_, the side chain of Lys-22 was protected with an allylcarboxycarbonyl (Aloc) group. Prior to the last Fmoc deprotection Lys-22 was orthogonally deprotected by treatment with tetrakis(triphenylphosphine)palladium(0) (Pd(PPh_3_)_4_) (347 mg, 0.3 mmol) in a mixture of chloroform:acetic acid:morpholine (85:10:5 v/v/v) for 2 hours in a N_2_ atmosphere. The deprotected peptides were washed sequentially with DIPEA (30 mM in DMF) and diethyldithiocarbamic acid (20 mM in DMF), followed by DMF and DCM. The resin was resuspended in DCM and 3-azidopropionic acid (115 mg, 1 mmol), *N*-Hydroxysuccinimide (NHS) (115 mg, 1 mmol), 1-Ethyl-3-[3-(dimethylamino)propyl]-carbodiimide (EDC) (191 mg, 1 mmol), and DIPEA (436 µl, 2.5 mmol) were added and allowed to react for 3 hours to attain JR2EK-Az and JR2EK_ref_-Az. The N-terminal Fmoc group was removed by treatment with piperidine in DMF and JR2EK/JR2EK_ref_ were cleaved from their solid support by exposure to a solution of trifluoroacetic acid (TFA), water and triisoproylsilane (95:2.5:2.5v/v/v) for 2 hours followed by filtration and evaporation of the solvent. JR2EC was cleaved using an alternative cleavage cocktail composed of TFA, ethanedithiol, water and triisoproylsilane (94:2.5:2.5:1 v/v/v/v). The peptides were precipitated twice in cold diethylether and purified using a gradient of aqueous isopropanol containing 0.1% TFA on an ACE-5 C-8 column attached to a semi-preparative high-performance liquid chromatography (HPLC) system (Dionex) (Figure S12). Post-synthetic modifications and the identity of the purified peptides were confirmed by matrix-assisted laser desorption/ionization time of flight mass spectrometry (MALDI-TOF MS) (Applied Biosystems) in linear positive mode using α–cyano-4-hydroxycinnamic acid (CHCA) as matrix (Figure S13).

### HA-BCN synthesis

Hyaluronic acid (200 mg, 0.5 mmol, Mw 100–150 kDa, Lifecore Biomedical) was dissolved in MES buffer (20 ml, 100 mM, pH 7.0) and agitated for 2 hours before *N*-[(1R,8S,9S)-Bicyclo[6.1.0]non-4-yn-9-ylmethyloxycarbonyl]-1,8-diamino-3,6-dioxaoctane (BCN-NH_2_) (50 mg, 0.15 mmol) dissolved in 5 ml acetonitrile: water (5:1 v/v), 1-Hydroxybenzotriazole hydrate (HOBt) (42 mg, 0.3 mmol) and EDC (118 mg, 0.6 mmol) was added. The reaction was allowed to proceed for 24 hours and HA-BCN was exhaustively dialyzed (MW cutoff 12–14 kDa, Spectra/Por RC, Spectrum Laboratories Inc.) in a mixture of MQ-water and acetonitrile (10:1 v/v) followed by MQ-water and finally lyophilized yielding HA-BCN with a degree of modification of ~7% based on ^1^H-NMR (Figure S1).

### HA-JR2EK/JR2EK_ref_ synthesis

HA-BCN (52 mg, 0.1 mmol) was suspended in MES buffer (15 ml, 10 mM, pH 10.0) for 15 minutes until fully dissolved. JR2EK-Az alternatively JR2EK_ref_-Az (63 mg, 0.015 mmol) were dissolved in MES buffer (10 ml, 100 mM, pH 7.0) and was added to the HA-BCN solution and the pH adjusted to 7.0. The reaction was allow to proceed for 24 hours and the derivatized HA was exhaustively dialyzed (MW cutoff 12–14 kDa, Spectra/Por RC, Spectrum Laboratories Inc.) in MQ-water and finally lyophilized yielding HA-JR2EK/JR2EK_ref_ with a degree of modification of ~4% based on ^1^H - NMR signals for the amino acid residues histidine and phenylalanine (Figure S1).

### JR2EC-AuNP synthesis

Gold nanoparticles (AuNPs) (20 nm, Cline Scientific AB) were functionalized with the peptide JR2EC as described previously^32, 36^.

### Enzyme encapsulation and hydrogel disassembly and degradation

HA-JR2EK (50 µl, 2.5 wt %) in Bis-Tris buffer (30 mM, pH 7.0) and JR2EC-AuNPs (2.5 µl, ~100 nM in Bis-Tris buffer) were added to a semi-macro cuvette and mixed before Zn^2+^ (ZnCl_2_, 5 µl, 100 mM in Bis-Tris buffer) was added. The cuvette was centrifuged to ensure that the formed gel where only distributed at the bottom of the cuvette (Figure S10). Bis-Tris buffer with and without Zn^2+^ (5 mM) was added to the cuvette yielding a final volume of 1 ml. UV-vis measurements were commenced and the cuvette was rapidly shaken on an orbital shaker in-between measurements. For the enzymatically catalyzed degradation experiments, freshly prepared hyaluronidase (5 µl, 0.025–2.5 mg/ml, 0.4–40 µM in Bis-Tris buffer) was added prior to addition of Zn^2+^ when casting the hydrogels or into the buffer after casting. After the last measurement 6 mM EDTA was added to completely dissemble the hydrogel to define the maximum peak intensity for JR2EC-AuNP, which was used for the normalization and calculations of percentage dissociation/degradation.

### Reactive oxygen species scavenging

Neutrophil granulocytes were isolated from human whole blood according to previously described protocols^37^. Samples were run in at least duplicates and the number blood donors were 2–3, depending on treatment. Cells (1 × 10^6^/mL in HEPES buffer) were loaded with 2′,7′-Dichlorofluorescein diacetate (DCF-DA) (5 µM) for 30 minutes prior to addition of test substances (HA-JR2EK (2.5 wt %) assembled using Zn^2+^ (10 mM), Bis-Tris buffer were used in control experiments) and phorbol 12-myristate 13-acetate (PMA) (1 µM). Fluorescence was monitored with an Infinite M1000 Pro plate reader (Tecan) (excitation wavelength of 485 ± 10 nm and emission wavelength of 530 ± 20 nm) during 80 minutes. Statistical significance was evaluated using paired t-test (GraphPad Prism ver 6.07).

### Characterization

All measurements were performed in Bis-Tris buffer (30 mM, pH 7.0) at room temperature unless otherwise noted. Dynamic light scattering (DLS) measurements were carried out in prefiltered (0.2 µm pore size, VWR) buffers at 21.5 °C on an ALV/DLS/SLS-5022F system (ALV GmbH), using a HeNe laser at 632.8 nm with 22 mW output power. The signal was detected perpendicular to the laser and each experiment was averaged of 10 runs of 30 s each. Circular dichroism (CD) spectra were acquired using a Chirascan^TM^ spectropolarimeter (Applied Photophysics) using a 0.1 mm cuvette. Each CD experiment was run in triplicates, averaged and background subtracted. Nuclear magnetic resonance (NMR) spectra were recorded on a Varian instrument (^1^H 300 MHz) in D_2_O with an additive of NaOD. Chemical shifts were assigned with the D_2_O peak as reference. Rheology experiments were carried out on a Discovery HR-2 rheometer (TA instruments) using a 20 mm 1° cone plate working in oscillatory mode. Frequency sweeps were measured at a fixed strain of 1% and amplitude sweeps at a frequency of 1 Hz. Gel recovery was evaluated at 1% strain and 1 Hz for 30 min after exposure to 1000% strain at 1 Hz for 180 s. Probe tack tests were carried out using a 8 mm steel plate-probe. The sample volume was 20 µl and the probe was lowered to a fix distance of 250 µm. After 60 s of incubation the probe was retracted at a constant speed of 100 µm/s and the resulting changes in the axial normal force was measured. The SEM measurements were performed on a LEO 1550 Gemini (Zeiss) operating at 5 kV. Samples containing 2.5 wt % HA-JR2EK with and without 10 mM Zn^2+^ were fixated with 1% glutaraldehyde over night at 4 °C. The samples were dehydrated by incubation in increasing concentrations of ethanol and finally in hexamethyldisilazane and were sputter coated with Pt prior to imagine. The UV-vis measurements were performed on a UV-2450 spectrophotometer (Shimaduzu) and absorbance spectra were recorded with a resolution of 0.5 nm. ATR FTIR measurements were performed on a Vertex 70 (Bruker Corp) with 750 scans for each sample and background subtracted with a sample of Bis-Tris buffer.

## Electronic supplementary material

Supplementary Information

Supplementary movie 1

Supplementary movie 2
