# Supplementary material for: Folding driven self-assembly of a stimuli-responsive peptide-hyaluronan hybrid hydrogel
Source: Sci Rep. 2017 Aug 1;7:7013. doi: 10.1038/s41598-017-06457-9 (PMC5539109; doi:10.1038/s41598-017-06457-9)
Supplement: Supplementary file 1 — Supplementary Information [file 41598_2017_6457_MOESM1_ESM.doc]

Supporting information

Folding driven self-assembly of a stimuli-responsive peptide-hyaluronan hybrid hydrogel

Robert Selegård,^1^ Christopher Aronsson,^1^ Caroline Brommesson,^2^ Staffan Dånmark,^1^ and Daniel Aili*^1^

*^1^Division of Molecular Physics, Department of Physics, Chemistry and Biology. Linköping University, SE-583 36, Linköping, Sweden*

**daniel.aili@liu.se*

*^2^Division of Molecular Surface Physics & Nanoscience, Department of Physics, Chemistry and Biology. Linköping University, SE-583 36, Linköping, Sweden*

Table of Contents

[Figure S1. 3](#__RefHeading___Toc484087480)

[Figure S2. 4](#__RefHeading___Toc484087481)

[Figure S3. 5](#__RefHeading___Toc484087482)

[Figure S4. 6](#__RefHeading___Toc484087483)

[Figure S5. 7](#__RefHeading___Toc484087484)

[Figure S6. 8](#__RefHeading___Toc484087485)

[Figure S7. 9](#__RefHeading___Toc484087486)

[Figure S8. 10](#__RefHeading___Toc484087487)

[Figure S9. 11](#__RefHeading___Toc484087488)

[Figure S10. 12](#__RefHeading___Toc484087489)

[Figure S11. 13](#__RefHeading___Toc484087490)

[Figure S12. 14](#__RefHeading___Toc484087491)

[Figure S13. 15](#__RefHeading___Toc484087492)

[References 16](#__RefHeading___Toc484087493)


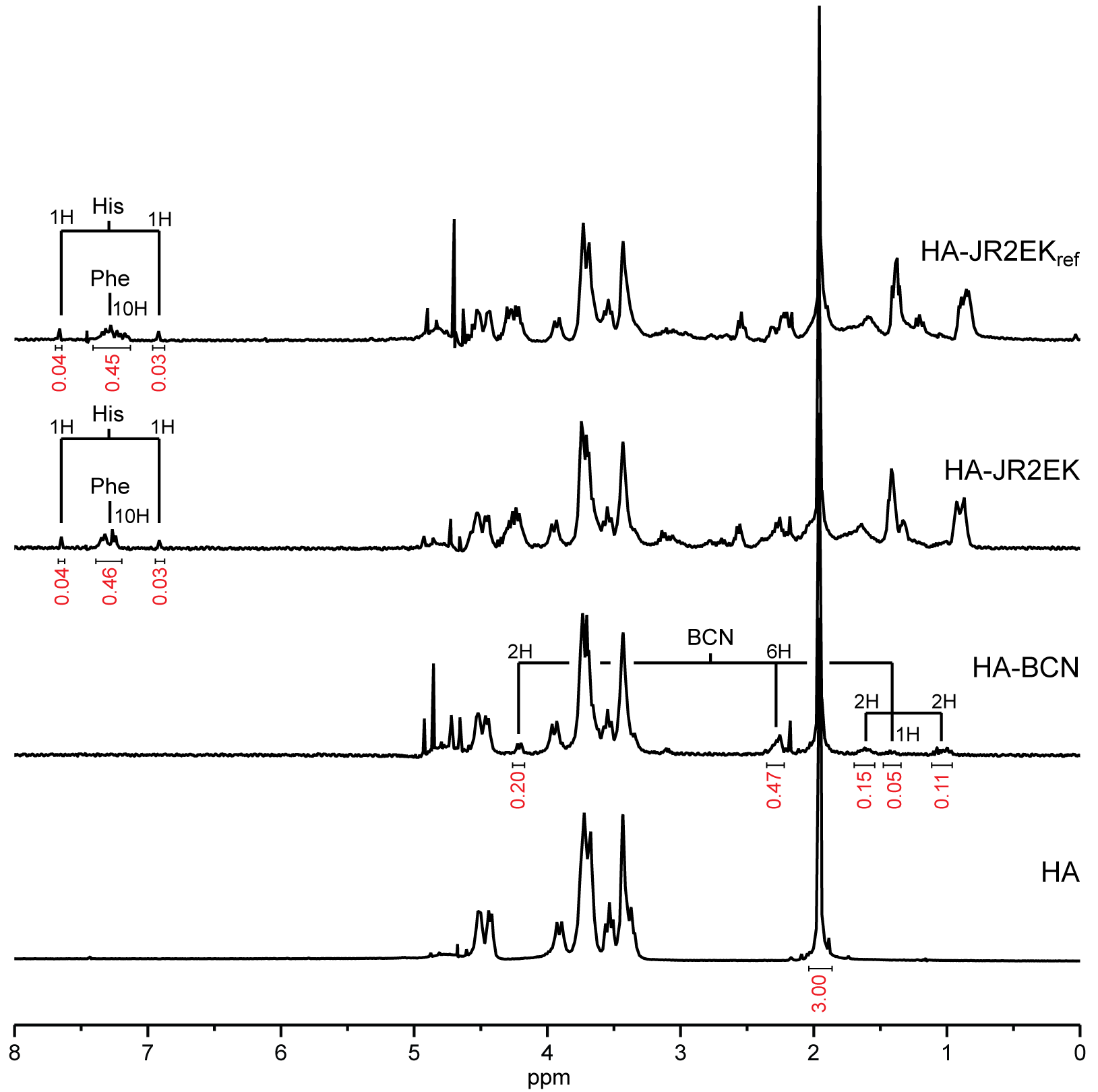


Figure S1. ^1^H-NMR spectra of HA, HA-BCN, HA-JR2EK and HA-JR2EK_ref_ recorded in D_2_O with peaks used for assessment of degree of functionalization marked. All integrals were normalized with respect to peak deriving from *N*-acetyl (3H) in HA at 1.95 ppm. Peaks derived from the BCN-group in HA-BCN was compared to unreacted BCN-NH_2_ (spectrum not shown, also with ^1^H-NMR data from J. Dommerholt et al.)^1^ and based on averaging of marked integrals with respect to the number of hydrogens, a degree of functionalization of ~7% was calculated. The aromatic residues histidine (one residue in peptide, 2 S*1H) and phenylalanine (two residues in peptide, m, 10H) were used for calculating the degree of functionalization for HA-JR2EK (~4%) and HA-JR2EK_ref_ (~4%).


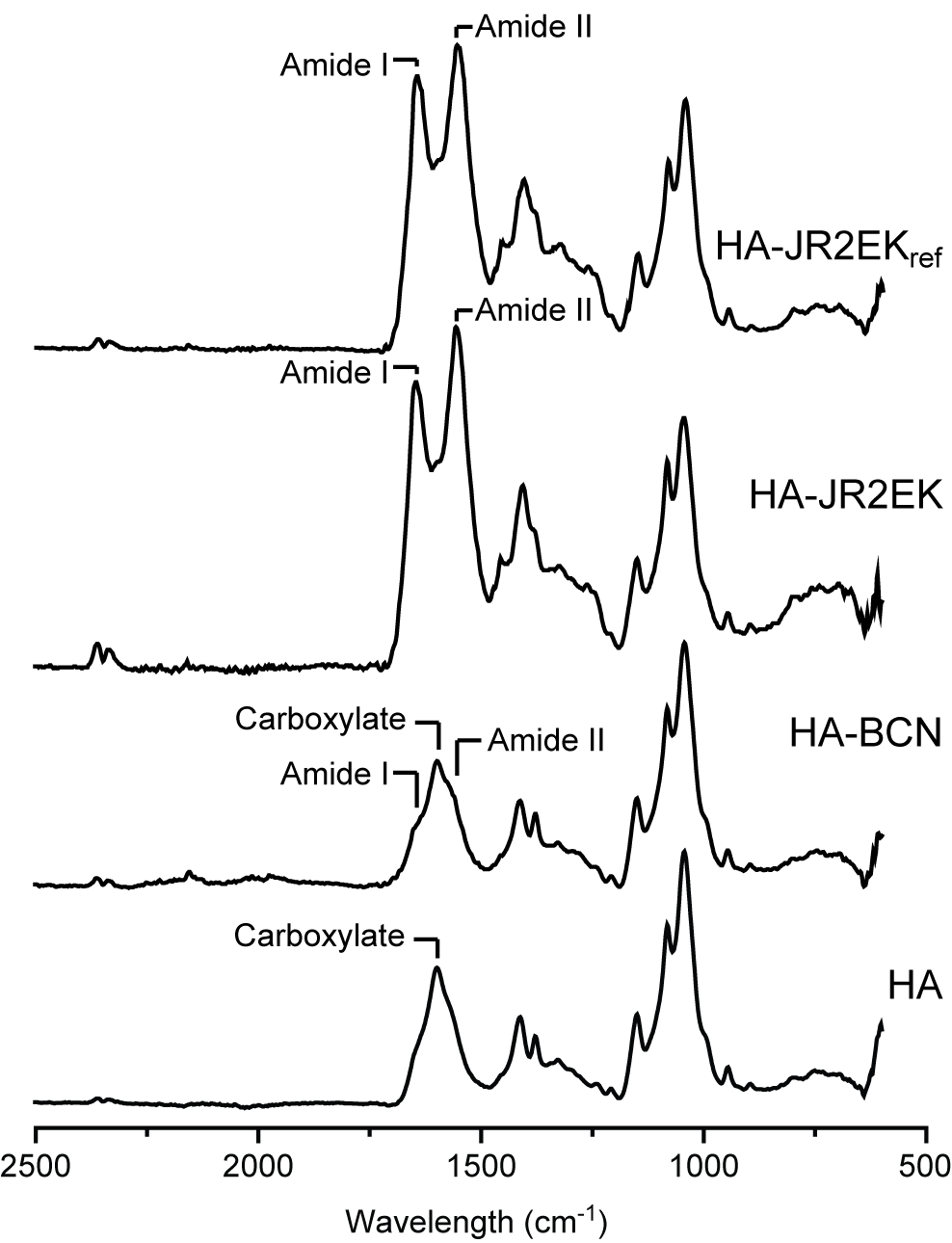


Figure S2. FT-IR spectra of HA, HA-BCN, HA-JR2EK and HA-JR2EK_ref_ with amide I (1643 cm^-1^), amide II (1556 cm^-1^) and the carboxylate (1600 cm^-1^) peaks indicated. The intensity of the carboxylate peak in HA-BCN is reduced compered to HA and broaden due to the overlap with amide I and II peaks. In the spectra of HA-JR2EK and HA-JR2EK_ref_ the amide I and II peaks from the amide bonds in the peptide backbone clearly stands out.

**
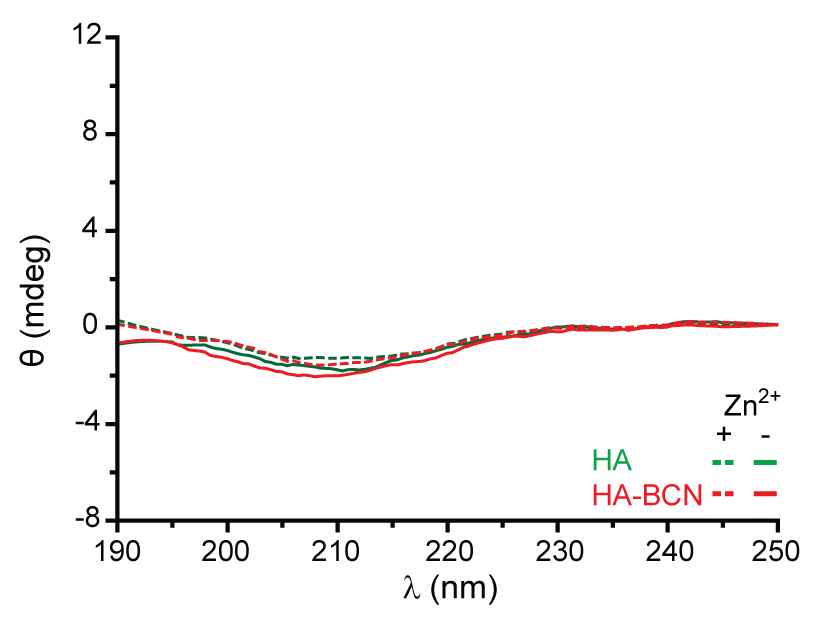
**

Figure S3. CD spectra of 0.1 wt % HA (green) and HA-BCN (red) with (dashed) and without (solid) 10 mM Zn^2+^.


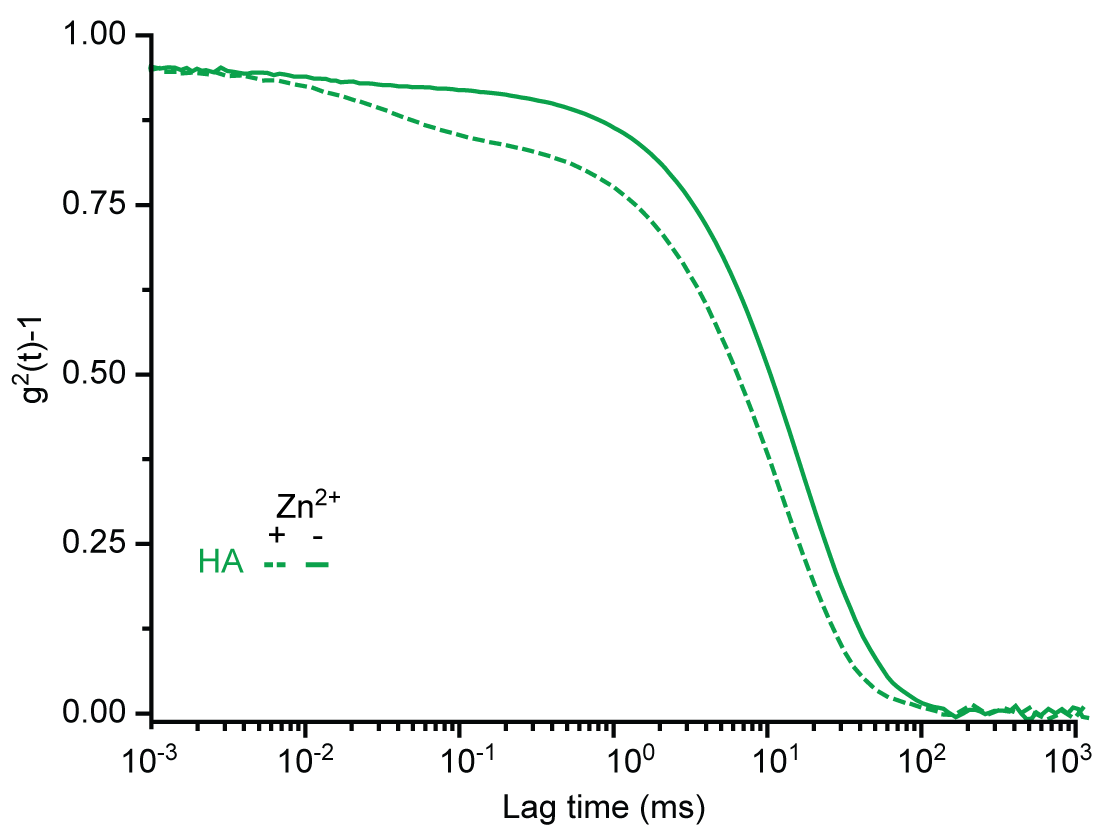


Figure S4. Normalized autocorrelation functions of 1.0 wt % of HA with (dashed) and without (solid) 10 mM Zn^2+^.


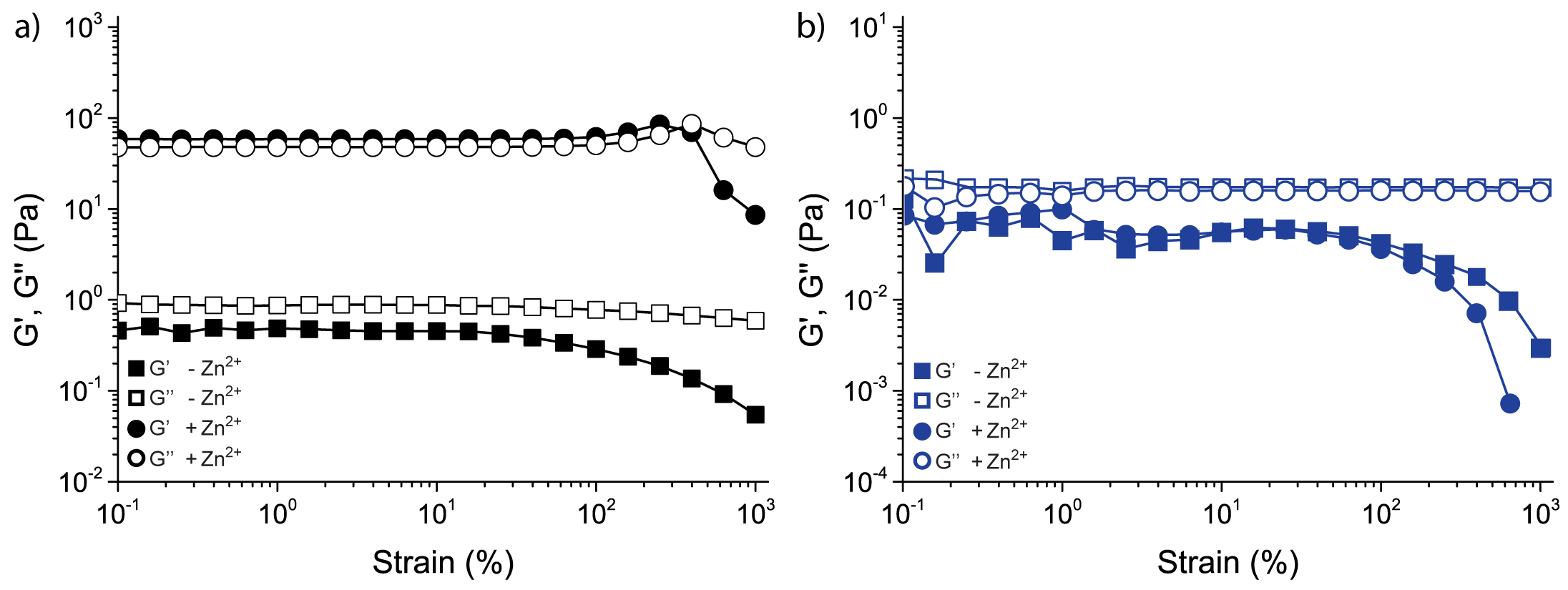


Figure S5. Strain sweeps of 2.5 wt % of HA-JR2EK (a) and HA-JR2EK_ref_ (b) with (circular) and without (square) 10 mM Zn^2+^.

**
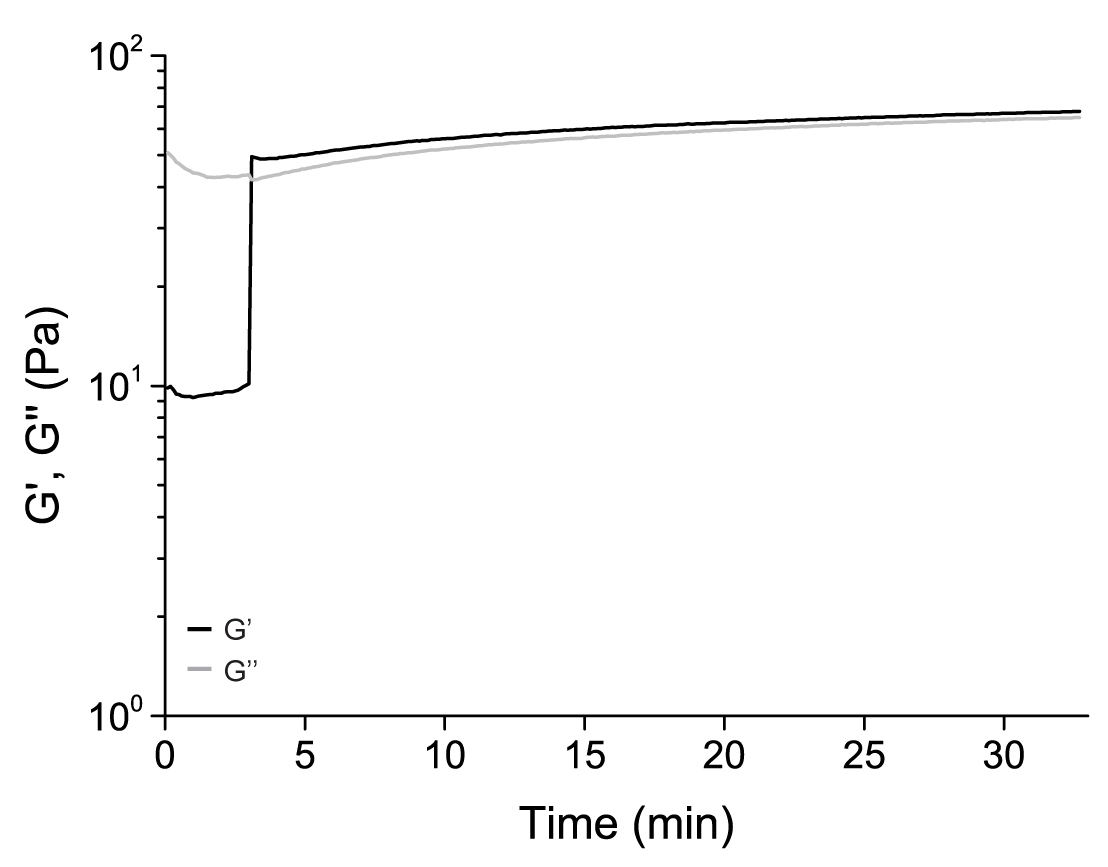
**

Figure S6. Gel recovery over time of 2.5 wt % HA-JR2EK self-assembled with 10 mM Zn^2+^ after excess mechanical strain (1000% strain, 1 Hz) for 180 s. After removing the excess mechanical strain the hydrogel recovered its G’ almost instantaneously.

**
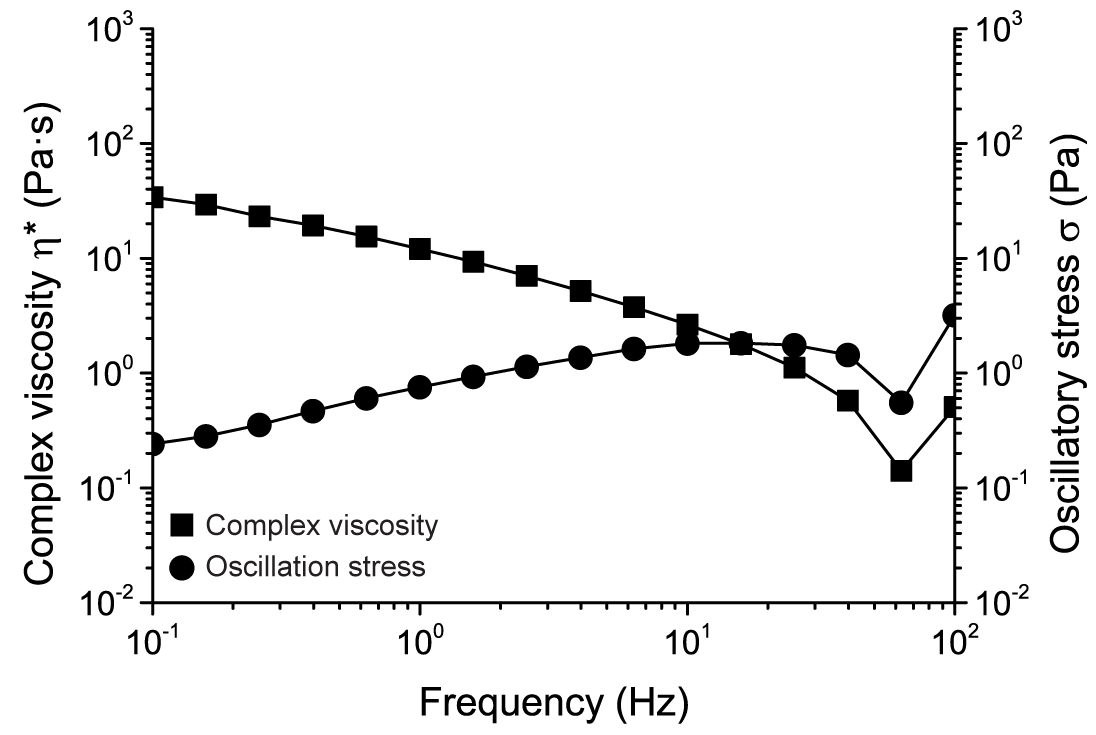
**

Figure S7. Frequency sweep showing the shear-thinning behaviour of 2.5 wt % HA-JR2EK with 10 mM Zn^2+^.


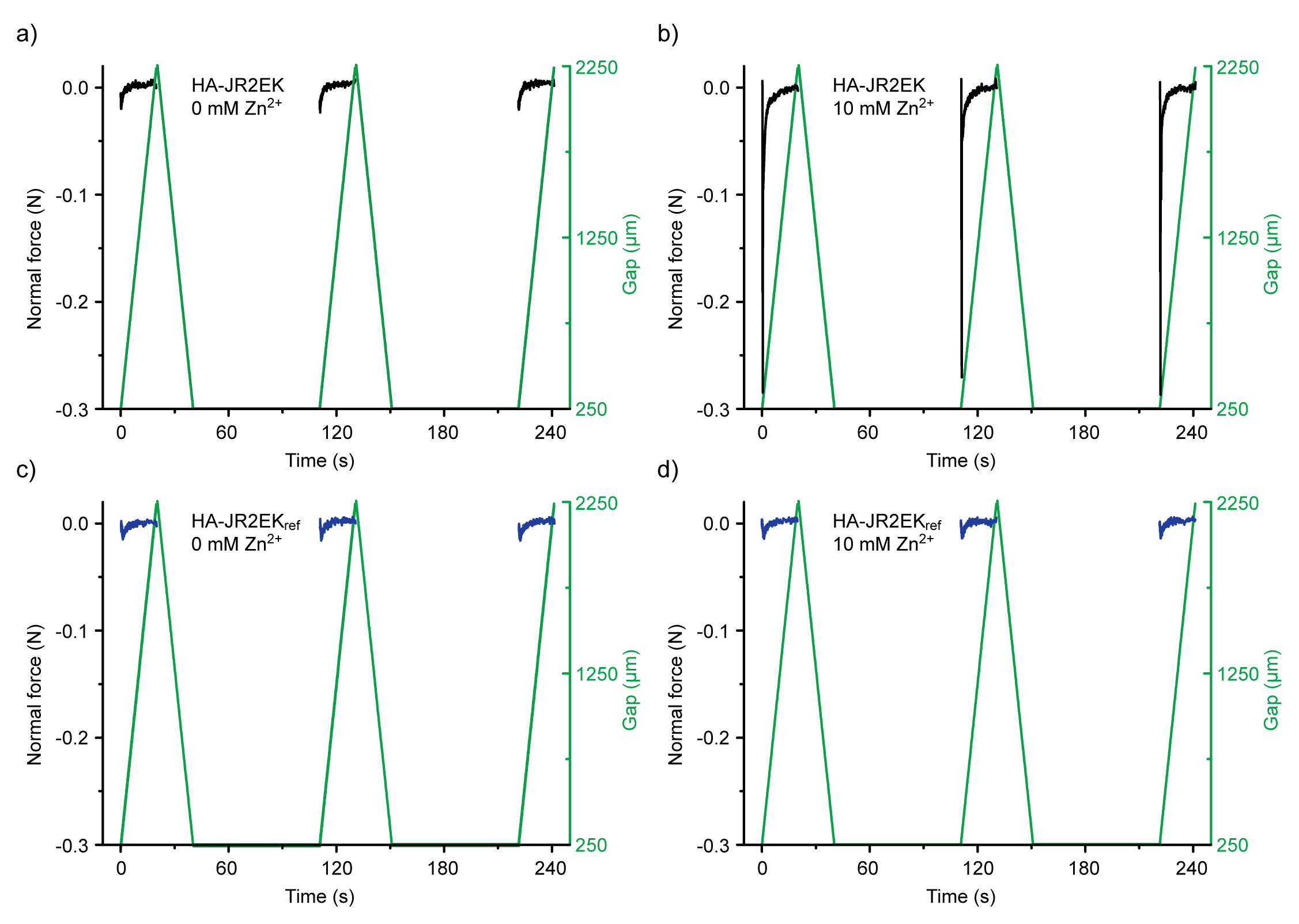


Figure S8. Repetitive probe tack tests of 2.5 wt % HA-JR2EK and HA-JR2EK_ref_ with and without 10 mM Zn^2+^.


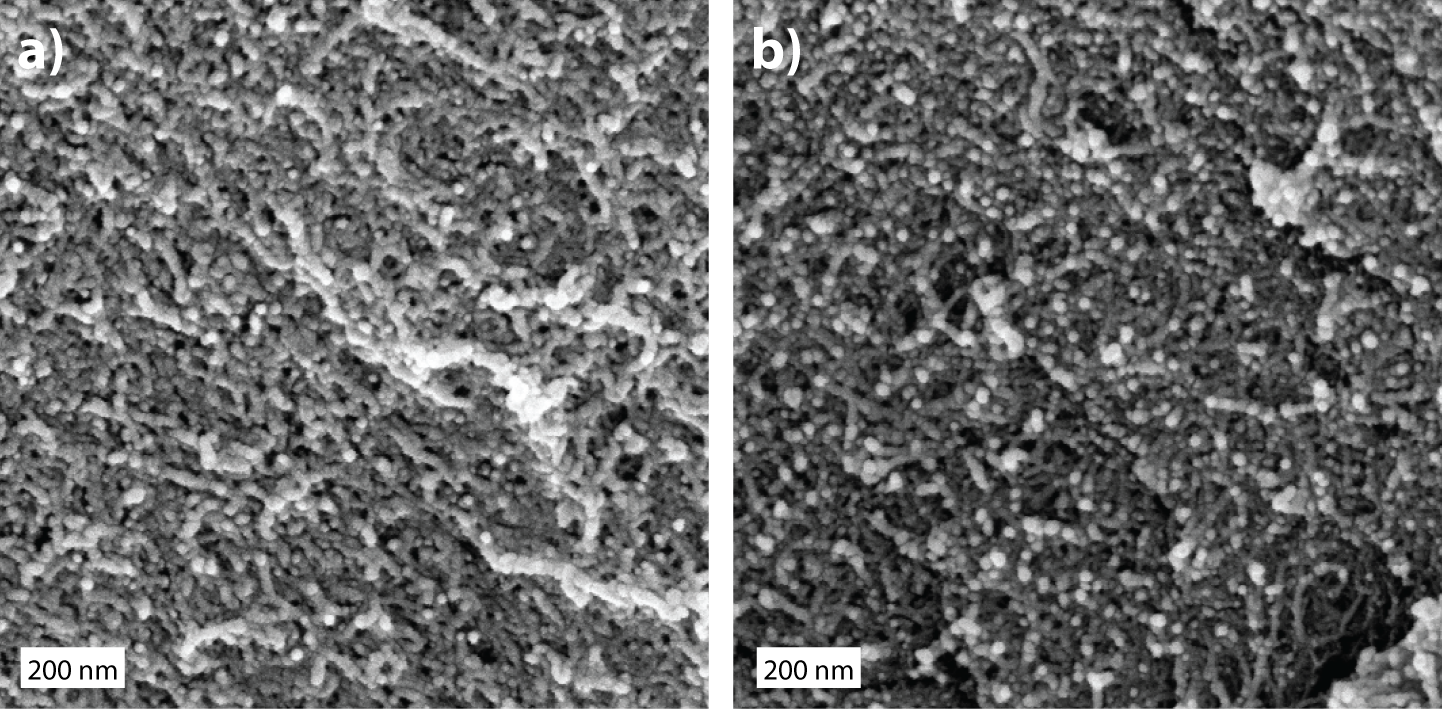


Figure S9. Scanning electron micrographs of 2.5 wt% HA-JR2EK with (a) and without (b) 10 mM Zn^2+^. Samples were fixated using 1 % glutaraldehyde.


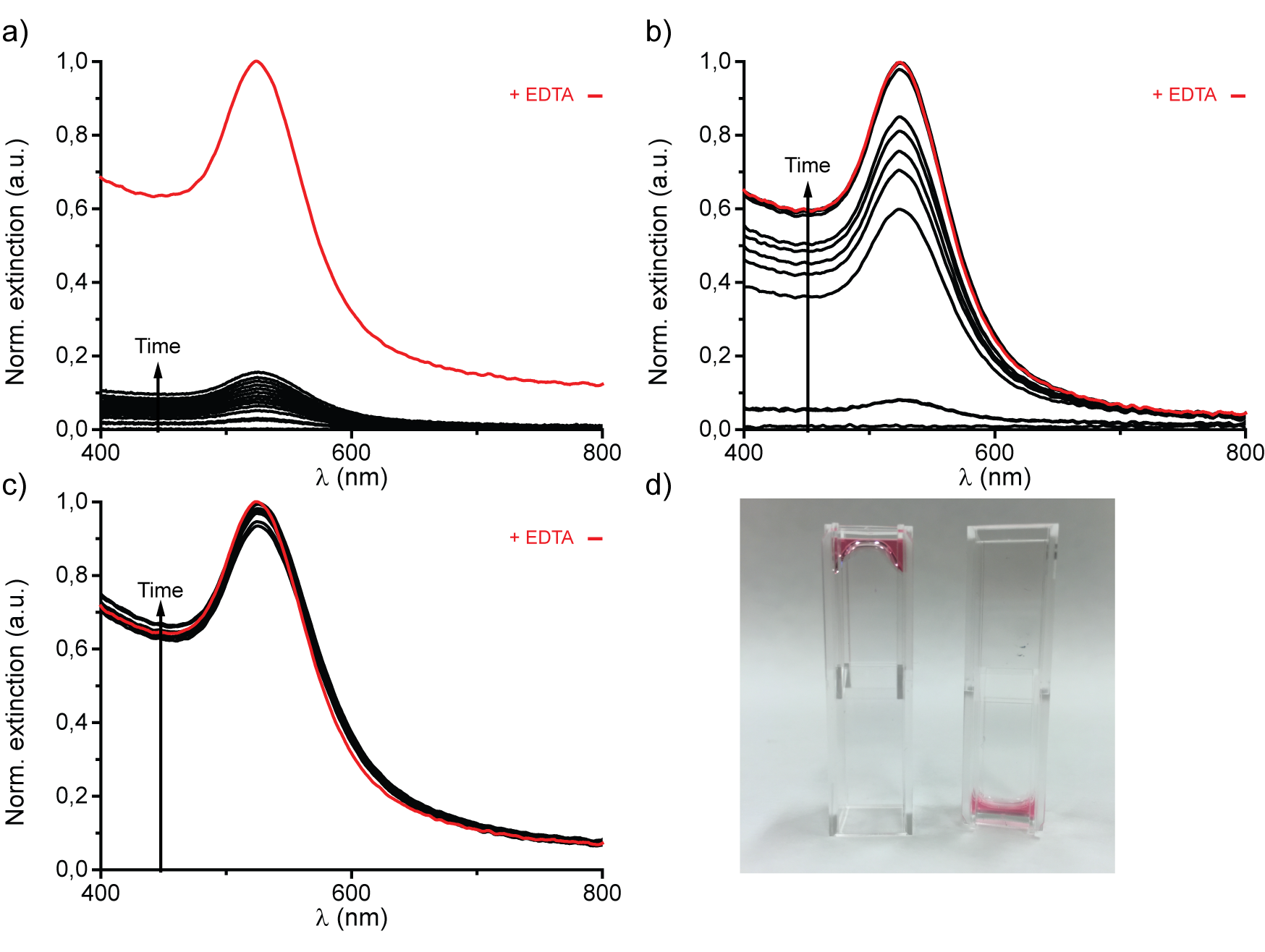


Figure S10. UV-vis spectra of 2.5 wt % HA-JR2EK with encapsulated JR2EC-AuNPs submerged in a buffer containing 5 mM (a) and 0 mM (b) Zn^2+^. c) UV-vis spectra of 2.5 wt % HA-JR2EK_ref_ with encapsulated JR2EC-AuNPs submerged in a buffer containing 5 mM Zn^2+^. UV-vis spectra were recorded every 30 minutes for 7 hours. Red curves indicate maximal degradation of the hydrogel, achieved by adding 6 mM EDTA. d) Photographs of 2.5 wt % HA-JR2EK hydrogels containing encapsulated JR2EC-AuNPs treated with 10 mM Zn^2+^ and casted in UV-vis cuvettes.


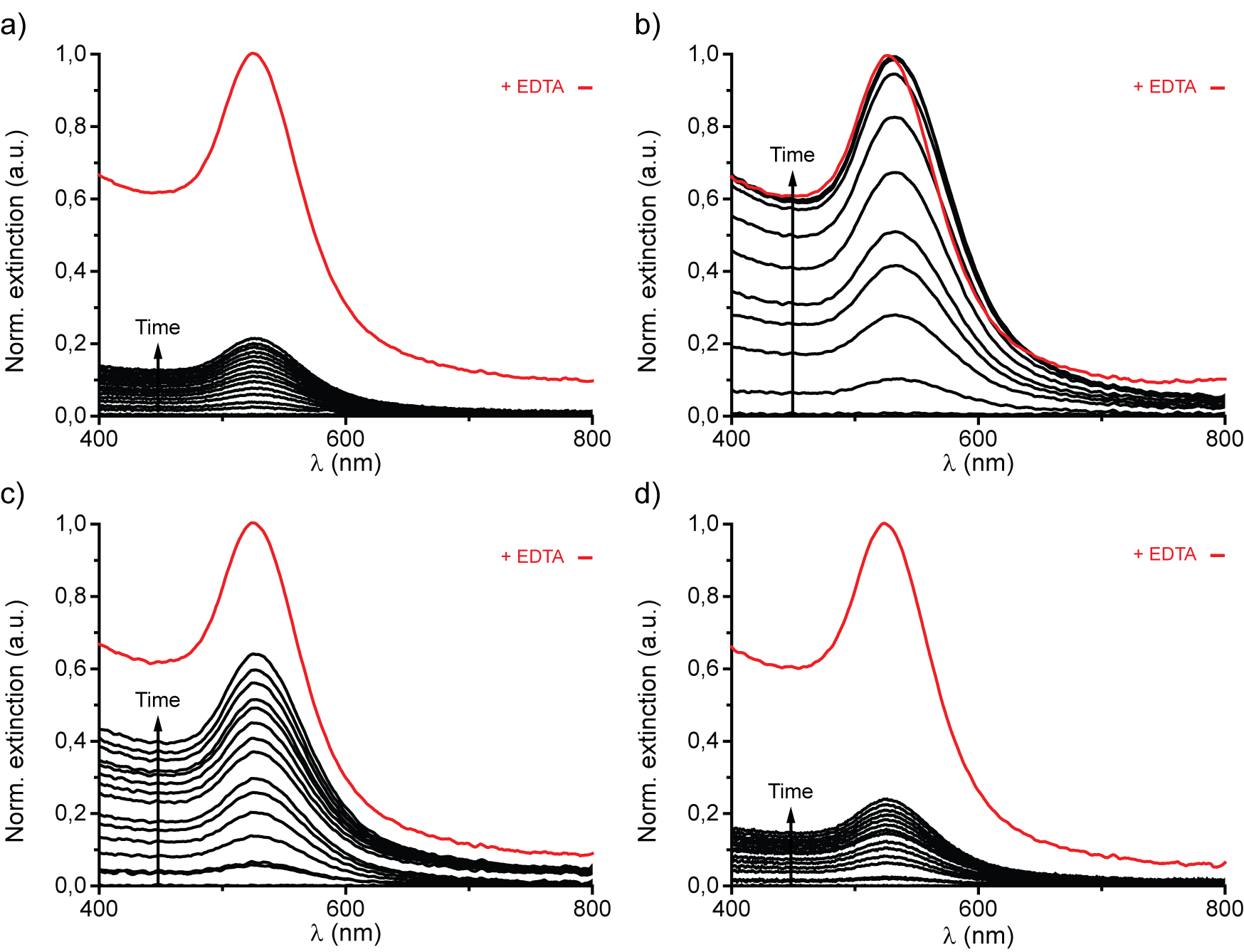


Figure S11. UV-vis spectra of encapsulated JR2EC-AuNPs released due to enzymatic degradation of 2.5 wt % HA-JR2EK with a) 200 nM hyaluronidase in the buffer and b) 200 nM c) 20 nM d) 2 nM hyaluronidase entrapped with in the hydrogel. UV-vis spectra were recorded every 30 minutes for 7 hours. Red curves indicate full dissociation of the hydrogels, achieved by addition of 6 mM EDTA. All experiments were carried out in buffer containing 5 mM Zn^2+^.


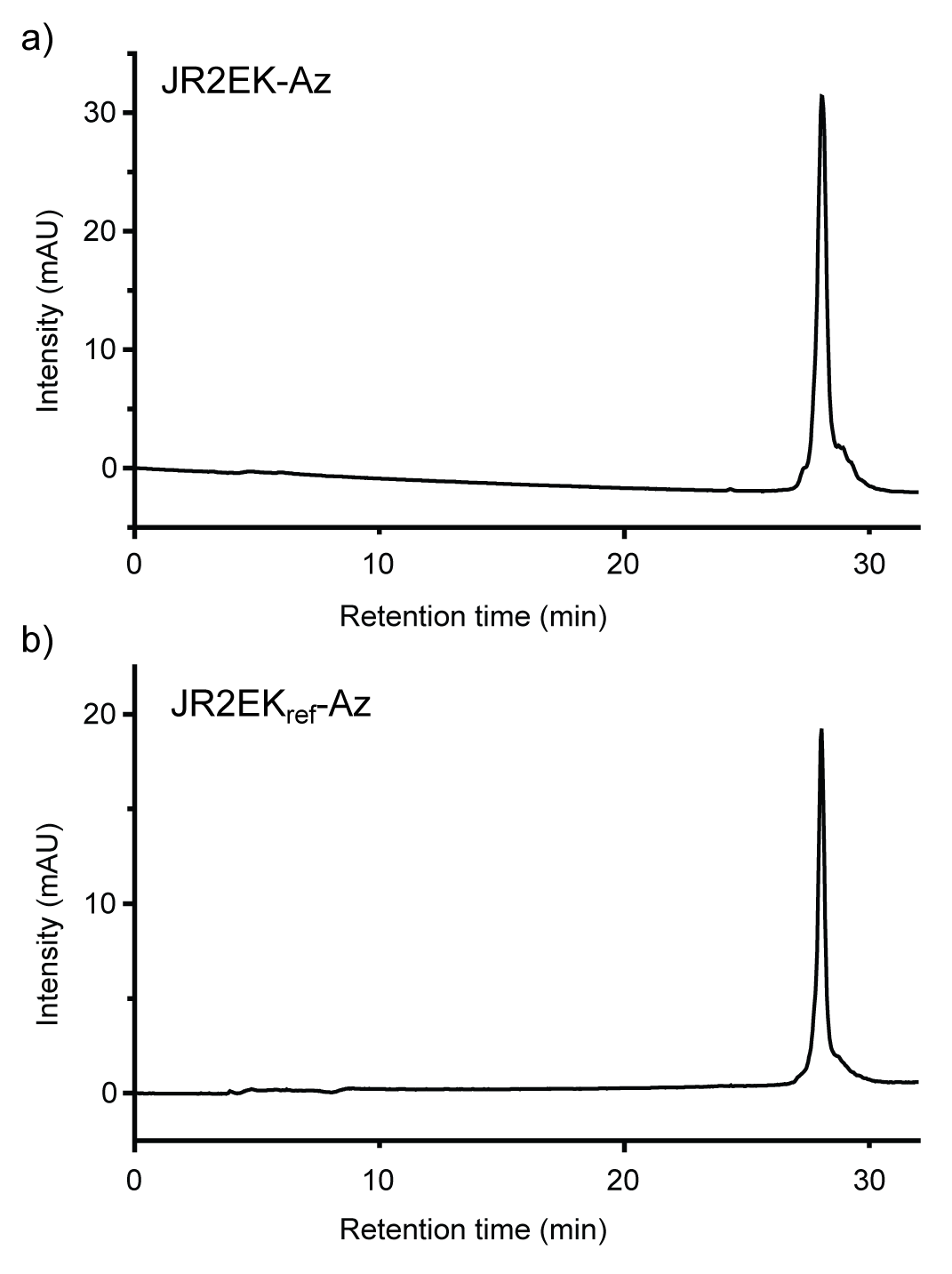


Figure S12. HPLC traces of purified a) JR2EK-Az and b) JR2EK_ref-_Az in an aqueous gradient of acetonitrile (10-50%) with 0.1% TFA.


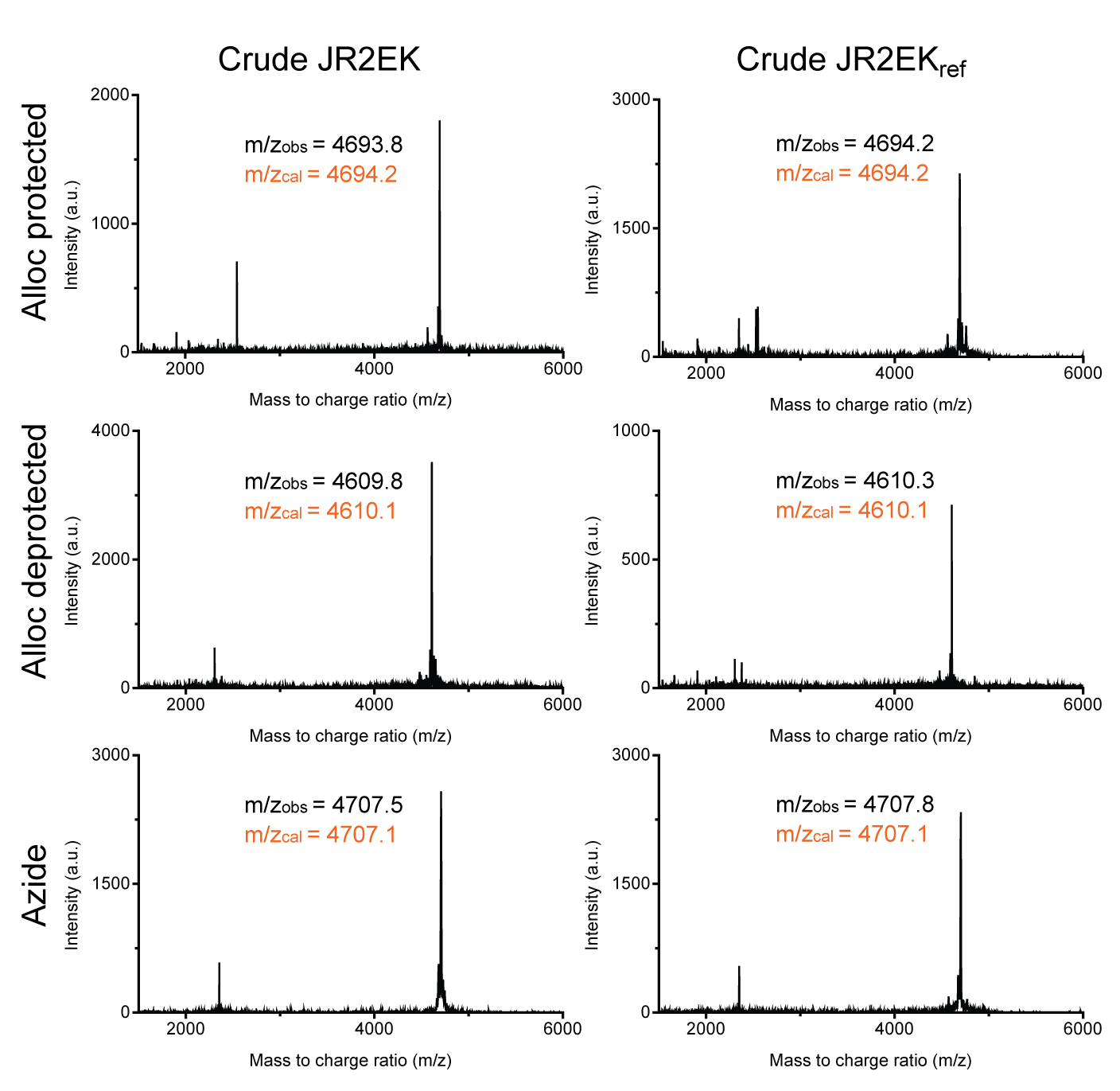


Figure S13. Mass spectra of JR2EK and JR2EK_ref_ before and after removal of the aloc protection group and after derivatization with 3-azidopropionic acid. A small amount of resin was treated with TFA after each reaction to enable MS-analysis of the crude peptides.

# References

[1] J. Dommerholt et al.; Angew. Chem., Int. Ed. 2010, 49, 9422-9425.
